# A biomimetic nanoplatform enables shTHY1-mediated immunomodulation and cartilage remodeling for osteoarthritis therapy

**DOI:** 10.1016/j.mtbio.2026.103145

**Published:** 2026-04-21

**Authors:** Xinyue Hu, Zhuang Li, Xiaofei Li, Lingxiao Zhang, Yaqing Zhang

**Affiliations:** a Department of Pediatric Orthopedics, Xin Hua Hospital Affiliated to Shanghai Jiao Tong University School of Medicine, Shanghai, PR China; b School of Medicine, Southeast University, Nanjing, Jiangsu, PR China; c The Orthopedics Department, Jinhua Municipal Centeral Hospital, Jinhua Hospital of Zhejiang University Medical College, Jinhua, Zhejiang Province, PR China; d Interdisciplinary Nanoscience Center, Aarhus University, Aarhus C, 8000, Denmark

**Keywords:** Osteoarthritis, shTHY1, Macrophage membrane, ROS, Biomimetic system, Immunomodulatory nanoparticles

## Abstract

Osteoarthritis (OA) is characterized by a chronic inflammatory microenvironment accompanied by elevated reactive oxygen species (ROS), synovial immune dysregulation, and progressive cartilage degeneration. While conventional pharmacological treatments often exhibit limited efficacy in halting disease advancement, we report a biomimetic, ROS-responsive nanoparticle (NPs) delivery system coated with macrophage membranes (MM-shTHY1-NPs) to achieve targeted therapy. The macrophage membrane (MM) coating not only improves retention and targeting toward inflamed synovial tissues, but also provides cytokine-scavenging capability that helps buffer the inflammatory microenvironment. Within the oxidative joint microenvironment, the ROS-responsive architecture of the NPs—facilitated by diselenide bonds—triggers the site-specific release of shTHY1 payloads. Importantly, we demonstrated the therapeutic relevance of THY1 silencing in OA microenvironment remodeling; both in vitro and in vivo experiments demonstrate that shTHY1 delivery effectively promotes macrophage repolarization from a pro-inflammatory M1 to an anti-inflammatory M2 phenotype. This phenotypic shift subsequently remodels the chondrocyte microenvironment, suppressing matrix metalloproteinase expression and attenuating cartilage degradation. The synergy between RNAi-mediated THY1 silencing and the cytokine-scavenging capability of the macrophage membrane coating resulted in enhanced therapeutic efficacy. Our findings suggest that this cell-membrane-coated nanoplatform provides a promising strategy for the treatment of inflammatory joint diseases, with potential advantages in biosafety, site-specific delivery, and translational feasibility.

## Graphical abstract

**Figure:**
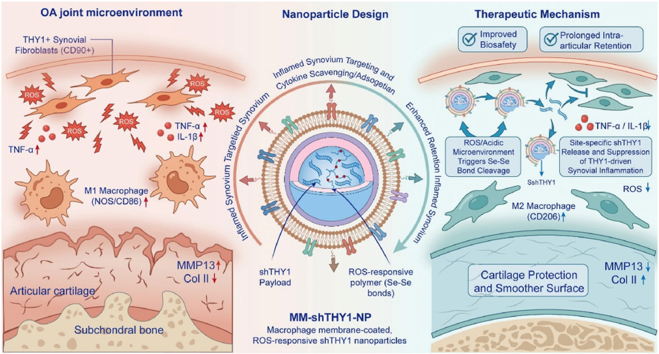

## 1 Introduction

Globally, 240 million people suffer from symptomatic and functionally limiting osteoarthritis (OA), significantly impacting patients' quality of life and socioeconomic conditions, which has garnered worldwide attention [1]-[2]. OA, as a chronic disease, is clinically characterized by persistent inflammation, degenerative changes in joint cartilage structure, and subsequent osteophyte formation [3]. Currently, the primary clinical treatments for OA include nonsteroidal anti-inflammatory drugs (NSAIDs), corticosteroids, and hyaluronic acid-based adjunctive therapies [4]-[5]. Although these treatments can effectively relieve pain and inflammation, they do not fundamentally halt the progression of OA [6]-[7]. Furthermore, long-term use of NSAIDs and corticosteroids can lead to a series of adverse reactions [8]. Therefore, there is an urgent need for further research into the mechanisms of OA to develop effective therapeutic strategies [9]-[10].

As the basic unit of life, cells constantly interact and exchange substances with their surrounding environment, and they can accumulate in certain specific microenvironments [11]. This insight is significant for the development of biomimetic drugs. Biomimetic drug delivery systems, particularly those involving cell membrane-coated nanoparticles (NPs), have garnered extensive attention from healthcare professionals [12]. Tasciotti reported the first leukocyte membrane-coated nanoparticles, which could prolong drug circulation time and enhance tumor accumulation [13]. Zhang described NPs coated with dual cell membranes from red blood cells (RBCs) and neutrophils, used for treating arthritis [14]. As immune cells, macrophages play a crucial role in the physiological environment of the human body and are closely related to the occurrence and development of various inflammatory pathologies. Notably, macrophages also play a significant role in the progression of OA [15]. At different stages of inflammation, various pathogenic stimuli or adverse microenvironments can induce the polarization of macrophages towards either the M1 or M2 phenotype, thereby achieving specific functions [16]-[17]. During the progression of OA, M1 macrophages stimulate the catabolic activity of chondrocytes, leading to the degradation of the extracellular matrix (ECM). Conversely, M2 macrophages enhance the anabolic activity of chondrocytes, exerting a protective effect. Therefore, coordinating macrophage polarization, specifically shifting from the pro-inflammatory M1 phenotype to the anti-inflammatory M2 phenotype, represents a practical strategy for the treatment of OA.

However, the polarization state of macrophages is not an isolated phenomenon but is precisely orchestrated by diverse cellular components within the synovial microenvironment [18]. Recently, the heterogeneity of synovial fibroblasts (SFs) and their pivotal role in OA have emerged as a significant research focal point [19]. Recent studies suggest that THY1^+^/CD90^+^ sublining fibroblasts are associated with inflammatory stromal states and exhibit a pro-inflammatory and invasive phenotype [20,21]. These fibroblasts engage in reciprocal crosstalk with synovial macrophages through cytokines and chemokines such as IL-6, TNF-α, IL-1β, and CCL2, thereby sustaining synovitis and promoting macrophage polarization toward the pro-inflammatory M1 phenotype [22]. This stromal–immune interaction not only amplifies intra-articular oxidative stress, but also accelerates extracellular matrix (ECM) degradation, including type II collagen loss, through upregulation of matrix metalloproteinases (MMPs). Therefore, we hypothesized that RNAi-mediated THY1 silencing could attenuate THY1-associated pro-inflammatory activation of synovial fibroblasts, disrupt fibroblast–macrophage inflammatory crosstalk, reduce M1-polarizing cues in the synovial microenvironment, and ultimately achieve chondroprotection [23].

**Scheme. 1:**
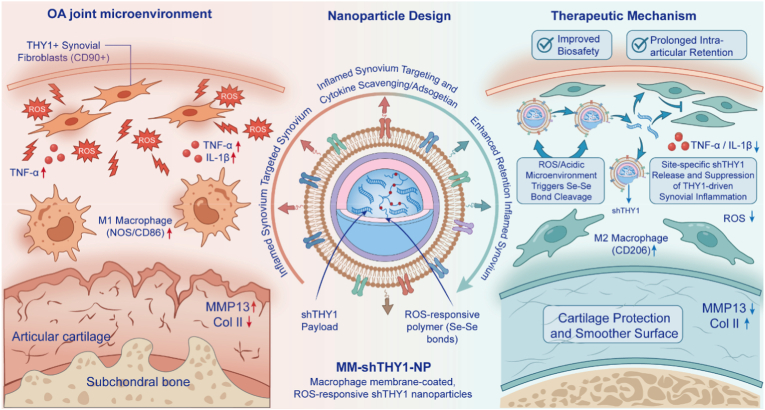
Schematic illustration of macrophage membrane–coated, ROS-responsive shTHY1 nanoparticles (MM-shTHY1-NPs) for osteoarthritis (OA) therapy. In the osteoarthritic joint, excessive ROS and inflammatory cytokines activate THY1^+^ (CD90^+^) synovial fibroblasts and promote pro-inflammatory M1 macrophage polarization, accelerating cartilage matrix degradation (MMP13↑, Col II↓). MM-shTHY1-NPs leverage macrophage membrane camouflage to enhance targeting and cytokine scavenging within inflamed synovium, while the ROS/acidic microenvironment triggers Se–Se bond cleavage and nanoparticle degradation to enable on-demand, site-specific release of shTHY1. The delivered shTHY1 suppresses THY1-driven synovial inflammation, reprograms macrophages from M1 to reparative M2 (CD206↑), reduces TNF-α/IL-1β and ROS, and ultimately protects cartilage with improved biosafety and prolonged intra-articular retention.

In this study, we developed a reactive oxygen species (ROS)-responsive nanoparticle (NP) delivery system coated with macrophage membranes (MMs) (abbreviated as MM-NPs) for the treatment of OA [24,25]. First, we designed ROS-responsive NPs formed by the self-assembly of long-chain polymers. Through a simple two-step amidation reaction, we synthesized the HA-SA-SD polymer long chain—consisting of hyaluronic acid (HA), stearic acid (SA), and selenocystamine dihydrochloride (SD)—and encapsulated short hairpin RNA targeting THY1 (shTHY1) within the particles to form shTHY1-NPs. Due to the presence of diselenide bonds (Se-Se bonds) in the SA-SD segments, the polymer undergoes degradation at pathological sites with elevated ROS levels and low pH, thereby accelerating drug release. Diselenide bonds possess lower bond dissociation energy than disulfide bonds and are therefore more susceptible to oxidative cleavage. In the presence of H_2_O_2_, Se–Se bonds can be oxidized into higher-valence selenium-containing groups, resulting in increased hydrophilicity and reduced structural stability of the polymer segments, which ultimately promotes nanoparticle disassembly and shTHY1 release. The outer macrophage membrane (MM) not only enhances the targeted delivery efficiency of NPs and their payloads to the diseased sites through biomimetic effects but also acts as a scavenger for pro-inflammatory factors, neutralizing the inflammatory microenvironment (Scheme 1). Subsequently, we conducted an in-depth investigation into the in vivo therapeutic efficacy of the MM-shTHY1-NP system for OA treatment.

## 2 Materials and methods

### 2.1 Materials

Shanghai National Pharmaceutical Group supplied hyaluronic acid (HA), stearic acid (SA), selenocystamine dihydrochloride (SD), DMSO, hydrogen peroxide (H_2_O_2_), and N-hydroxysuccinimide (NHS). Anti-iNOS (K010080P), anti-CD206 (K006619P), CCK-8 kit (CA1210), and ROS detection kit (CA1410) were obtained from Solarbo (China). All remaining chemicals and reagents were of analytical quality.

### 2.2 Preparation of MM-sh THY1-NPs

#### 2.2.1 Preparation of ROS-responsive sh THY1-NPs

1) Dissolve 300 mg of stearic acid in 30 mL of anhydrous DMSO. Subsequently, add 300 mg of EDC dissolved in 6 mL of DMSO to the SA solution and activate at room temperature for 30 min. Next, add 200 mg of SD dissolved in 10 mL of DMSO to the activated SA solution and react at room temperature for 6 h. Dialyze and lyophilize.
2) Dissolve 100 mg of HA in 5 mL of MES buffer. Then, add 100 mg of EDC dissolved in 0.5 mL of MES buffer to the HA solution and stir at room temperature for 1 h. Slowly drip 100 mg of SD-SA dissolved in 30 mL of DMSO into the activated HA solution and react at room temperature for 8 h. After 8 h, precipitate with acetone, wash three times, and dry the product under vacuum for 30 min. Disperse the powder in distilled water, dialyze, and lyophilize.

#### 2.2.2 Preparation of biomimetic macrophage MM-sh THY1-NPs

Macrophage membranes were prepared as previously reported [26]. In summary, RAW264.7 cells were resuspended in a buffer solution at 4 °C to a concentration of 2.0 × 10^6^ cells/mL and subsequently lysed with a mini extruder. The cell extract was combined with 1 M sucrose to achieve a final concentration of 0.25 M, then spun at 2000×*g* for 10 min at 4 °C. The supernatant was collected and centrifuged again at 3000×*g* for 30 min. To purify, the cell membranes obtained were rinsed twice with a chilled TM buffer solution containing 0.25 M sucrose. The overall protein quantity in the isolated macrophage membranes was measured with the bicinchoninic acid (BCA) protein test.

### 2.3 Characterization of sh THY1-NPs and MM-sh THY1-NPs

The zeta potential and particle size of sh THY1-NPs and MM-sh THY1-NPs under different concentrations of H_2_O_2_ were measured using NanoZetasizer and the morphology of the NPs was observed using SEM and TEM [27]. The stability of the nanoparticles was monitored by measuring the size and PDI changes of sh THY1-NPs and MM-sh THY1-NPs dispersed in PBS with pH 4.5 or pH 7.4 over one week at 25 °C. The in vitro release profile of sh THY1 was determined using a modified dialysis method. Briefly, the NPs were placed in a dialysis bag (MWCO: 3000 Da) and shaken at 100 rpm. Samples were collected at different hours for PCR analysis. WB was used to confirm the expression of macrophage and sh THY1. FTIR (iS10, Nicolet, USA) and UV-Vis (UV6100S, MAPADA, China) were used to detect characteristic functional groups. The encapsulation efficiency and loading capacity of shTHY1 in the nanoparticles were determined by quantifying the amount of free, non-encapsulated shTHY1 after nanoparticle preparation. Briefly, the nanoparticle suspension was transferred into an ultrafiltration tube (MWCO 100 kDa) and centrifuged at 5000 rpm for 15 min to separate free shTHY1 from nanoparticle-associated shTHY1. The filtrate containing free shTHY1 was collected and quantified using a Quant-iT RiboGreen RNA assay kit according to the manufacturer's instructions. A standard calibration curve was established using known concentrations of shTHY1. Then, the encapsulation efficiency (EE) and loading capacity (DL) were calculated.

### 2.4 Cell lines

iCell Bioscience Inc. provided RAW 264.7 cells, which resemble mouse macrophages, and Rat Cartilage Cells (RCC), derived from rat chondrocytes. Cells were grown in DMEM with 10% FBS and 1% PS at 37 °C in a 5% CO_2_ atmosphere.

The Thy1 knockdown vector was purchased from the Public Protein/Plasmid Library (PPL). The lentiviral packaging plasmids pVSVG, gag-pol, and rev were obtained from Nanjing BiYuntian Biotechnology. Plasmid information: The GenBank ID for Thy1 is 21838, and GFP serves as the reporter protein. The plasmid PPL50556-3a has the target sequence CCGCCATGAGAATAACACCAA, and the negative control target sequence is GTTCTCCGAACGTGTCACGT.

### 2.5 QT-PCR detection

mRNA levels were quantified using qRT-PCR. Macrophages were first placed in six-well plates and grown for a day until they were 70-80% confluent. After a 12-h exposure to LPS (1 μg/mL), different material groups were added and left to incubate for an additional 24 h. Total RNA was employed to extract total RNA following the supplier's instructions, and subsequently, a reverse transcriptase kit was used for reverse transcription. Cq values were determined using a real-time PCR system (Table S1).

### 2.6 In vitro cellular uptake assay

For the cellular uptake study, FITC was covalently conjugated to the amino-containing HA-SA-SD polymer through the isothiocyanate–amine reaction prior to nanoparticle self-assembly. The resulting FITC-labeled nanoparticles were then used to prepare MM-coated nanoparticles, and the fluorescence signal was used to trace nanoparticle internalization rather than the shTHY1 cargo itself. Similar to previous methods, macrophages were seeded in 12-well plates, then incubated with PBS, sh THY1, sh THY1-NPs, and MM-sh THY1-NPs at 37 °C. At specific time points, the cells were fixed with 4% paraformaldehyde, observed under a fluorescence microscope (Nikon, Japan), and the average fluorescence intensity of the images was quantitatively analyzed using ImageJ software.

### 2.7 In vitro cytocompatibility

The CCK-8 assay (CA1210, Solarbio, China) was used to evaluate the safety of MM-sh THY1-NPs on cells. Macrophages and RCC cells were plated at a concentration of 50,000 cells per well in 96-well plates. The cells were then incubated with sh THY1, sh THY1-NPs, and MM-sh THY1-NPs for 48 h. Afterward, the cells were treated with a 5 mg/mL CCK-8 solution and kept at 37 °C for 4 h. Ultimately, cell viability was determined by measuring the absorbance at 490 nm with a microplate reader (Infinite M200 Pro, Switzerland).

### 2.8 Intracellular ROS detection

The intracellular ROS levels were detected using a ROS detection kit. In short, rat chondrocytes (RCC) cells were planted in 24-well dishes. PBS, sh THY1, sh THY1-NPs, and MM-sh THY1-NPs were then added and incubated for 24 h. Subsequently, the medium was removed, and ROS working solution was added. Following a half-hour period, the cells were examined and photographed using a fluorescence microscope. ImageJ software was utilized to measure the typical brightness levels in the images.

### 2.9 Immunohistochemistry

For histological analysis, rat knee joints subjected to various treatments were collected for Hematoxylin and Eosin (H&E) staining, Masson staining, and Safranin O/Fast Green staining. For immunohistochemical analysis, the knee joint sections were incubated with primary antibodies (anti-MMP13 and anti-collagen) respectively.

### 2.10 Animal model

Male Sprague-Dawley (SD) rats (240-280 g) were purchased from the experimental animal center [28]. All animal studies were conducted in accordance with the university's animal experimentation guidelines and approved by the university's animal ethics committee. To create the OA model, the rats had ACLT performed on their right knees. As previously described, sham-operated rats were used as controls. The model was validated using the anterior drawer test. A week after surgery, ACLT rats were randomly assigned to five groups (n = 5) and given intra-articular injections of various formulations, while sham-operated rats acted as healthy controls. Each group of rats received intra-articular administration on days 1, 3, 5, and 7. All rats were euthanized on day 28, and joints were removed and collected for immunohistochemistry.

### 2.11 In vivo retention evaluation

Nine male SD rats (240-280 g) underwent ACLT on the right knee to establish the OA model, and a sham operation was performed on the left knee. Two months later, the retention capabilities of the formulations in healthy and OA knee joints were evaluated. Imaging of the rat knees was performed at 12, 24, 48, 72 and 120 h after injection utilizing an in vivo imaging system from Caliper Life Sciences, USA.

### 2.12 Statistical analysis

GraphPad Prism 8.0 (La Jolla, CA, USA) was utilized for all statistical computations. The statistical relevance was assessed through either a Student's t-test or a one-way ANOVA. The data are shown as the average ± standard deviation (SD), with a P-value of less than 0.05 indicating statistical significance.

## 3 Results and discussion

### 3.1 Preparation and characterization of MM-sh THY1-NPs

**Fig. 1:**
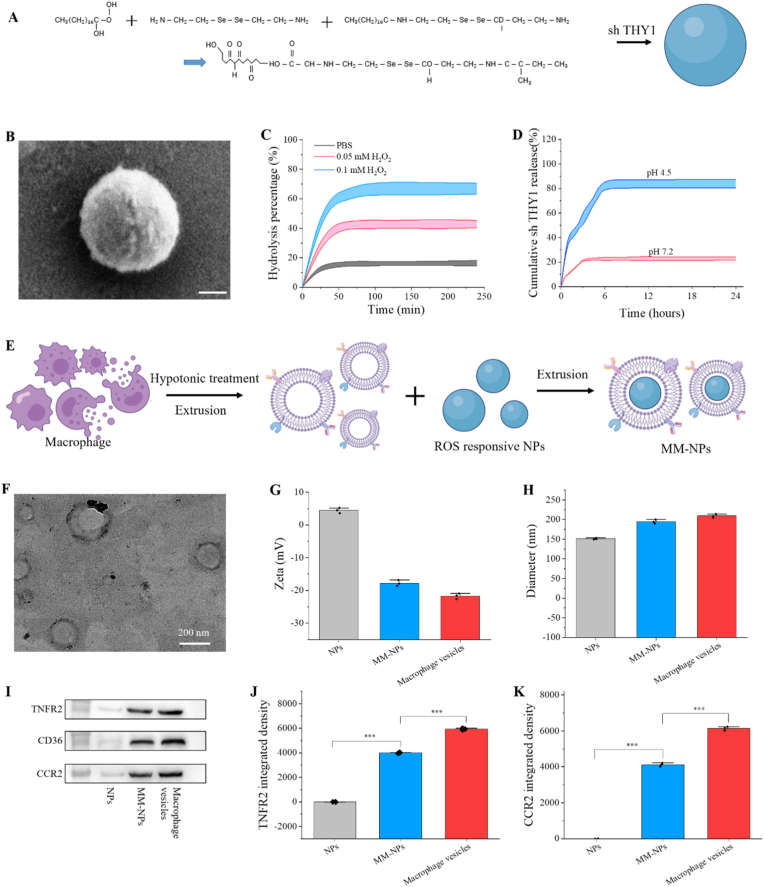
**Preparation and characterization of MM-sh THY1-NPs.** (A) Schematic illustration of NP preparation. (B) Representative SEM image of ROS-responsive NPs. Scale bar: 50 nm. (C) Hydrolysis rate of NPs at different H_2_O_2_ concentrations (0.50 mM and 0.1 mM). (D) In vitro drug release profile of sh THY1-NPs. (E) Schematic illustration of MM-NPs preparation via extrusion method. (F) Representative TEM image of MM-NPs. Scale bar: 200 nm. (G) Zeta potential and (H) particle size of NPs and MM-NPs analyzed by DLS. (I) Characteristic protein bands of NPs, macrophage membrane-derived vesicles, and MM-NPs separated by Western blot. (J) Quantitative analysis of TNFR2 and (K) CCR2 integral density in NPs, macrophages, and MM-NPs. All experiments were repeated five times (n = 5), and data are presented as mean ± standard deviation. Statistical analysis was performed using one-way ANOVA. ∗∗∗P < 0.001.

The ROS-responsive NPs were synthesized through the self-assembly of the amphiphilic polymer HA-SA-SD. The synthesis process of HA-SA-SD NPs is depicted in Fig. 1A. In aqueous solution, the amphiphilic HA-SA-SD polymer self-assembled into micelle-like nanoparticles, with stearic acid segments driving hydrophobic aggregation and HA chains forming the hydrophilic outer shell. During nanoparticle formation, negatively charged shTHY1 was incorporated through electrostatic interactions with the amino-containing SD segments, together with physical entrapment during self-assembly, rather than by simple partitioning into the hydrophobic core. The EE and DL of shTHY1 were determined to be 82.4 ± 2.7% and 6.3 ± 0.5%, respectively, indicating that the HA-SA-SD polymer could effectively load shTHY1 during nanoparticle self-assembly. Afterward, sh THY1-encapsulated nanoparticles were created using an uncomplicated self-assembly technique. Scanning electron microscopy (SEM) analysis revealed these NPs to be roughly spherical, with an average size of approximately 168 nm (Fig. 1B). UV and IR (Fig. S1) confirmed the successful synthesis of the HA-SA-SD polymer. To assess the reactivity of ROS, nanoparticles were exposed to different levels of hydrogen peroxide, and the transmittance of the solution was recorded at various intervals. Considering that ROS levels are persistently elevated in OA joints, whereas the instantaneous concentration of free H_2_O_2_ is difficult to define precisely due to dynamic local generation, diffusion, and antioxidant clearance, 0.1 mM H_2_O_2_ was used here as a disease-relevant oxidative trigger to simulate the enhanced oxidative stress microenvironment in OA. According to Fig. 1C, the particle mixture turned fully clear within 2 h when exposed to 0.1 mM hydrogen peroxide. Conversely, particles in PBS exhibited only a moderate decrease in absorbance over 4 h, attributed to the partial settling of the nanoparticles. Furthermore, we evaluated the release of the drug from sh THY1-NPs in various pH environments. As shown in Fig. 1D, sh THY1-NPs exhibited 82.5% drug release at pH 4.5, while only 21.3% drug release at pH 7.2, indicating excellent ROS responsiveness of the NPs. Not only that, we also investigated the size evolution of sh THY1-NPs under different pH conditions and H_2_O_2_ concentrations. As shown in Fig. S2–S3, the hydrodynamic diameter of sh THY1-NPs exhibited distinct time-dependent changes in response to acidic and oxidative environments, further confirming the stimulus-responsiveness of sh THY1-NPs. Although direct release characterization of MM-shTHY1-NPs was not separately performed in the present study, previous reports on macrophage/cell membrane-coated stimulus-responsive nanoplatforms suggest that membrane coating generally acts as a biomimetic outer shell and does not abolish the intrinsic responsiveness of the nanoparticle core. Instead, it may reduce premature leakage and modestly modulate release kinetics, while still allowing pathological microenvironment-triggered cargo release. To create MM-NPs, membranes from RAW264.7 murine macrophage cells were extruded to envelop the ROS-sensitive nanoparticles (Fig. 1E). Fig. 1F illustrates that MM-NPs displayed a spherical core-shell configuration under TEM, each nanoparticle being surrounded by a single-layer cell membrane with an approximate coating thickness of 9 nm, matching the cell membrane's thickness. According to DLS analysis, the zeta potential of MM-NPs was reduced compared to free NPs (Fig. 1G), yet it matched the zeta potential found on the macrophage surface. Additionally, DLS analysis revealed that the nanoparticle size grew from approximately 168 nm to around 207 nm following the application of the cell membrane coating (Fig. 1H), aligning with the incorporation of the macrophage bilayer membrane. To further determine whether macrophage membrane coating affects the microenvironment-responsive behavior of the nanoparticles, we additionally evaluated the hydrolysis and release profiles of MM-shTHY1-NPs under oxidative and acidic conditions. As shown in Fig. S4, MM-shTHY1-NPs still exhibited clear H_2_O_2_-responsive hydrolysis and pH-triggered shTHY1 release after membrane coating. These results indicate that macrophage membrane coating did not abolish the intrinsic stimulus-responsive behavior of the Se–Se-containing nanoparticle core, although the response kinetics were moderately attenuated due to the additional membrane barrier. Western blotting verified that essential membrane antigens like TNFR2, CD36, and CCR2 were present on boththe macrophage membrane and the surface of MM-NPs, demonstrating that the macrophage membrane on MM-NPs was like that on macrophages (Fig. 1I–K)^30^. Western blotting confirmed that key macrophage membrane proteins, including TNFR2, CD36, and CCR2, were retained on the surface of MM-NPs, indicating successful preservation of bioactive membrane components after the coating process (Fig. 1I–K). Importantly, these membrane-associated proteins are not merely structural markers but may also endow MM-NPs with functional anti-inflammatory properties. Specifically, TNFR2 may act as a decoy receptor to bind soluble TNF-α, thereby reducing its bioavailability and downstream inflammatory signaling. CCR2 may contribute to the sequestration of chemokines such as CCL2 in the inflamed microenvironment, which could help attenuate inflammatory cell recruitment and signal amplification. In addition, CD36, as a scavenger receptor, may facilitate the recognition and adsorption of oxidized lipids or damage-associated inflammatory cues, further buffering the local inflammatory burden. Therefore, the macrophage membrane coating in our system likely functions not only as a targeting shell, but also as a bioactive therapeutic interface that contributes to inflammatory mediator scavenging and microenvironment modulation. To further evaluate the cytokine-scavenging capability of the macrophage membrane coating, recombinant TNF-α and IL-1β were incubated with different nanoparticle formulations, and the remaining cytokine levels in the supernatant were quantified by ELISA. As shown in Fig. S5, MM-coated nanoparticles exhibited evident inflammatory cytokine-buffering activity. After 2 h incubation, the scavenging efficiencies of MM-NPs and MM-shTHY1-NPs reached 52.4 ± 3.8% and 57.9 ± 4.1% for TNF-α, respectively, and 38.7 ± 3.5% and 43.5 ± 3.9% for IL-1β, respectively. These findings provide direct quantitative evidence that the macrophage membrane coating contributes to pro-inflammatory cytokine scavenging, thereby synergizing with shTHY1 delivery to modulate the local inflammatory microenvironment. Simultaneously, the sh THY1 load successfully reduced intracellular THY1 expression. These results demonstrate the successful preparation of ROS-responsive macrophage-mimicking NPs loaded with sh THY1.

### 3.2 Biocompatibility and cellular uptake of MM-sh THY1-NPs

**Fig. 2:**
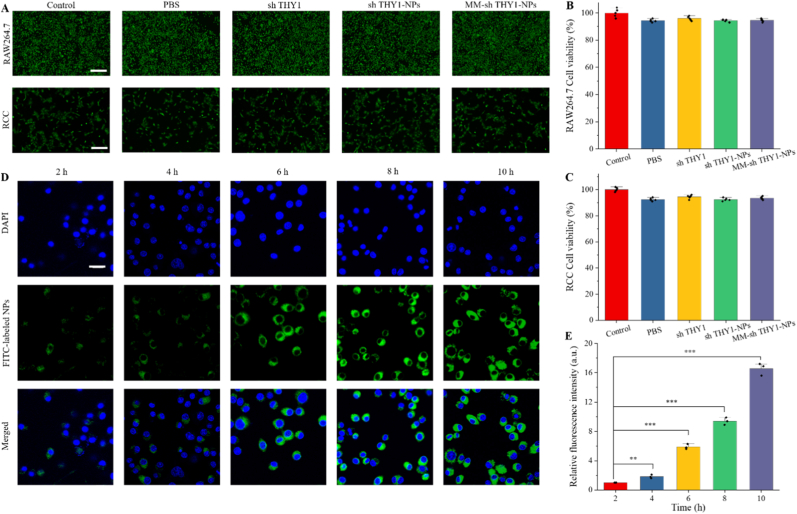
**Biocompatibility and cellular uptake of MM-sh THY1-NPs.** (A) Representative live/dead staining images of RAW264.7 macrophages and RCC chondrocytes co-cultured with different groups of materials for 24 h (green, live cells; red, dead cells). Scale bar, 200 μm. (B) and (C) Statistical analysis of cell viability of RAW264.7 macrophages and RCC chondrocytes, respectively, using CCK-8 assay (n = 3). (D) LSCM images at different time points of RAW264.7 macrophages co-cultured with FITC-labeled NPs, and (E) statistical analysis of fluorescence intensity (n = 3). Scale bar, 200 μm ∗∗P < 0.01; ∗∗∗P < 0.001.

Biocompatibility is a prerequisite for the efficacy of biomaterials in vivo. In this study, we evaluated the cytotoxicity of the materials using CCK-8 and live-dead staining assays. As shown in Fig. 2A, after co-culturing the materials with RAW264.7 cells and chondrocytes (RCC) for 48 h, most cells remained viable. Additionally, we assessed cell viability using the CCK-8 assay. As shown in Fig. 2B and C, after 48 h, cell viability in all material groups remained above 90%, with no significant difference compared to the control group (p > 0.05). This indicates that our materials exhibit good biocompatibility.

Next, we evaluated the cellular internalization behavior of different formulations in macrophages. As shown in Fig. 2D–E and Fig. S6, intracellular fluorescence increased progressively with incubation time, indicating a clear time-dependent uptake process. In addition, comparative analysis of different formulations showed that the intracellular fluorescence intensity in the MM-shTHY1-NPs group was significantly higher than that in the shTHY1-NPs group, suggesting that macrophage membrane coating enhanced macrophage internalization. Since a broader cell-selectivity assay was not performed, we described these results as enhanced macrophage uptake/internalization rather than direct evidence of macrophage-specific targeting. All representative images were standardized to the same magnification and display size to ensure valid comparison across groups.

### 3.3 Effects of MM-sh THY1-NPs on macrophage M1-M2 repolarization

**Fig. 3:**
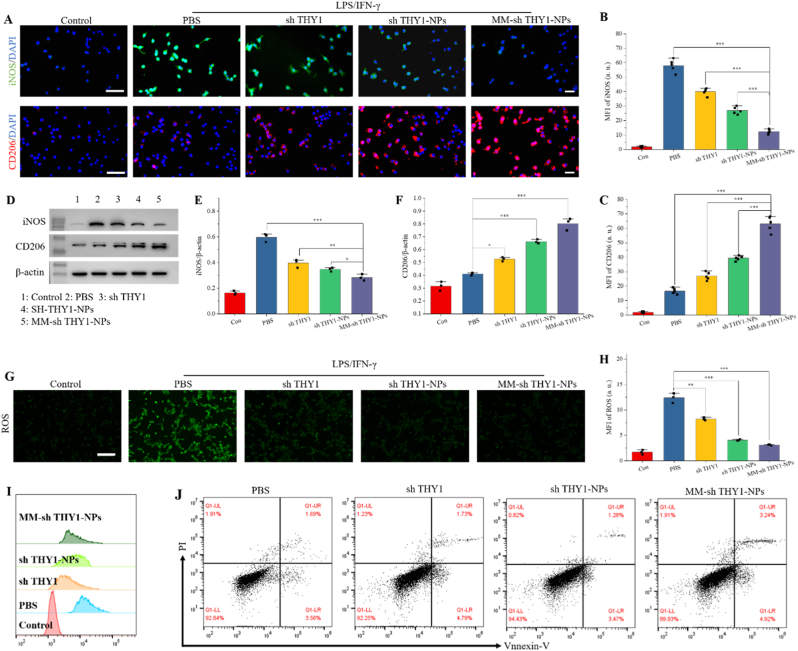
**Effect of MM-sh THY1-NPs on macrophage M1-M2 repolarization.** (A) Immunofluorescence images showing the expression of pro-inflammatory M1 biomarker (iNOS; green) and anti-inflammatory M2 biomarker (CD206; red) under various conditions. Scale bar, 100 μm. (B) and (C) Quantitative analysis of iNOS and CD206 expression under different conditions, respectively. (D) Western blot analysis showing the phenotypic shift of M1 (iNOS) to M2 (CD206) polarization after treatment with various materials, and quantitative analysis of iNOS (E) and CD206 (F). P < 0.05; ∗∗P < 0.01; ∗∗∗P < 0.001. (G) Immunofluorescence staining showing intracellular ROS levels in different groups, and (H) quantitative analysis (n = 3). Scale bar, 100 μm. (I) Flow cytometry analysis of intracellular ROS expression in different groups. (J) Flow cytometry analysis of Annexin V-FITC/PI ratio in different groups. ∗P < 0.05; ∗∗P < 0.01; ∗∗∗P < 0.001.

In classical inflammatory responses, macrophages act as effective effector cells, roughly divided into M1 and M2 subgroups to perform pro-inflammatory and anti-inflammatory functions, respectively, in response to different stimuli from their microenvironment. Before evaluating the effect of the nanoparticles on macrophage polarization, we first quantified the THY1 knockdown efficiency after shTHY1 treatment. As shown in Fig. S7, free shTHY1 significantly reduced THY1 mRNA expression compared with the PBS group, whereas nanoparticle-mediated delivery further enhanced this inhibitory effect. Notably, MM-shTHY1-NPs produced the strongest suppression of THY1 expression, confirming the effective gene-silencing capability of the RNAi nanoplatform. M1 macrophage activation can be induced by LPS or IFN-γ, while M2 macrophage polarization can be activated by IL-4 and/or IL-13. In OA joints, macrophages mainly exhibit an M1 phenotype, secreting large amounts of inflammatory cytokines. Therefore, we used LPS and IFN-γ to stimulate macrophages and evaluate the ability of MM-sh THY1-NPs to reprogram M1 macrophages. After LPS and IFN-γ induction, immunofluorescence staining primarily showed green fluorescence, indicating that macrophages were in the M1 state (Fig. 3A). Compared with the PBS group, the expression of M1 marker iNOS significantly decreased, and the expression of M2 marker CD206 significantly increased in the sh THY1, sh THY1-NPs, and MM-sh THY1-NPs treatment groups (Fig. 3B–C). Among them, the MM-sh THY1-NPs group had the lowest iNOS expression and the highest CD206 expression, suggesting that MM-sh THY1-NPs can promote macrophage M1 to M2 repolarization. We further detected M1 and M2 markers in macrophages using Western blot analysis. As shown in Fig. 3D–F, iNOS expression in the MM-sh THY1-NPs group was approximately 45% of the PBS group, and CD206 expression was 231% of the PBS group, further confirming that MM-sh THY1-NPs can promote macrophage M1 to M2 repolarization.

To clarify whether shTHY1 acts upstream at the fibroblast level, we first evaluated THY1 silencing efficiency in synovial fibroblasts. As shown in Fig. S8A, free shTHY1 significantly reduced THY1 mRNA expression compared with the PBS group, while shTHY1-NPs further enhanced this inhibitory effect. Notably, MM-shTHY1-NPs produced the most pronounced downregulation of THY1, indicating that the nanoparticle platform, particularly after macrophage membrane coating, improved THY1 silencing efficiency in synovial fibroblasts. We next examined whether suppression of fibroblast-associated THY1 signaling affected downstream macrophage polarization. As shown in Fig. S8B, shTHY1 intervention decreased iNOS expression and increased Arg1 expression under inflammatory conditions, with the strongest effect observed in the MM-shTHY1-NPs group. These findings suggest that silencing THY1 in synovial fibroblasts attenuates fibroblast-derived pro-inflammatory cues, thereby weakening M1-polarizing pressure and favoring macrophage transition toward a reparative M2 phenotype. Therefore, MM-shTHY1-NPs appear to remodel the OA synovial microenvironment at least in part by interrupting fibroblast–macrophage inflammatory crosstalk, which provides a mechanistic basis for the macrophage repolarization results shown below.

To further investigate the mechanism underlying macrophage repolarization, we examined NF-κB-related signaling by Western blot analysis. As shown in Fig. S9, shTHY1-based treatment altered the expression of p-p65 and IκBα, suggesting that the regulatory effect of the nanoplatform on macrophage polarization is at least partly associated with inhibition of NF-κB activation. These findings provide preliminary mechanistic support for the observed M1-to-M2 phenotypic shift.

### 3.4 Anti-inflammatory and chondroprotective effects of MM-sh THY1-NPs In vitro

Articular cartilage is primarily composed of chondrocytes and extracellular matrix (ECM). The ECM provides protection to chondrocytes, while chondrocytes regulate the synthesis and degradation of the ECM. Under normal conditions, chondrocytes secrete type II collagen and aggrecan, which are essential components of the ECM. During the progression of OA, oxidative stress and inflammatory factors cause chondrocytes to express collagen and matrix metalloproteinases, such as type II collagen and MMP-13. These proteins lead to the degradation of the extracellular matrix (ECM) and apoptosis of chondrocytes. In this study, we evaluated the anti-inflammatory and chondroprotective effects of MM-sh THY1-NPs in vitro by measuring the expression of inflammatory factors and marker proteins in cartilage synthesis and metabolism.

Macrophages and RCC cells were co-cultured and stimulated with LPS and IFN-γ to induce M1 differentiation, followed by the addition of sh THY1, sh THY1-NPs, and MM-sh THY1-NPs. Fig. 3G and H shows that ROS levels in the treatment groups decreased compared to the PBS group, with the best recovery observed in the MM-sh THY1-NPs group, which was 2.4 times that of the control group. Further quantification of cellular ROS using flow cytometry yielded consistent results, with the best recovery observed in the MM-sh THY1-NPs group (Fig. 3I), indicating that MM-sh THY1-NPs significantly reduce intracellular ROS. Annexin-V/PI analysis of chondrocyte apoptosis showed no significant difference in apoptosis rates between groups (p > 0.05, Fig. 3J and S10).

**Fig. 4:**
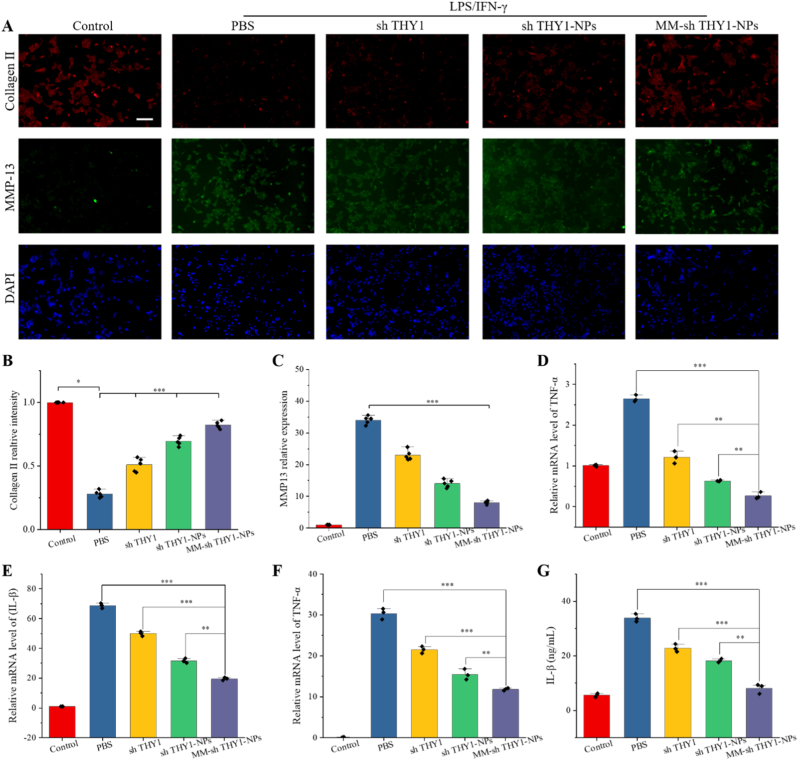
**Chondroprotective effects of MM-sh THY1-NPs in vitro.** (A) Immunofluorescence staining of chondrocyte growth and metabolism markers (green: MMP13, red: Collagen; blue: DAPI) and (B) quantitative analysis of Collagen fluorescence intensity, (C) quantitative analysis of MMP13 fluorescence intensity. Scale bar, 100 μm. (D) qRT-PCR analysis of inflammatory cytokine expression levels for TNF-α and (E) IL-1β.(F) ELISA quantification of inflammatory cytokine expression for TNF-α and (G) IL-1β in various treatment groups (N = 5). ∗P < 0.05; ∗∗P < 0.01; ∗∗∗P < 0.001.

In addition, we examined the expression of proteins related to cartilage synthesis and metabolism. As shown in Fig. 4A–C, collagen II expression significantly decreased, and MMP13 expression significantly increased in chondrocytes after LPS and IFN-γ stimulation. Compared to the PBS group, collagen II expression significantly increased, and MMP13 expression significantly decreased in the sh THY1, sh THY1-NPs, and MM-sh THY1-NPs treatment groups, with the best therapeutic effect observed in the MM-sh THY1-NPs group. PCR and ELISA further confirmed the expression of IL-1β and TNF-α expressions were significantly inhibited in the treatment groups (Fig. 4D–G). These results indicate that MM-shTHY1-NPs significantly alleviate inflammatory stress in chondrocytes and shift the chondrocyte microenvironment from a catabolic state toward a more anabolic state, as evidenced by decreased MMP13 and increased Collagen II expression. This beneficial transition is closely associated with macrophage repolarization from the pro-inflammatory M1 phenotype to the reparative M2 phenotype.

### 3.5 In vivo targeting ability and therapeutic effects of MM-sh THY1-NPs on OA

**Fig. 5:**
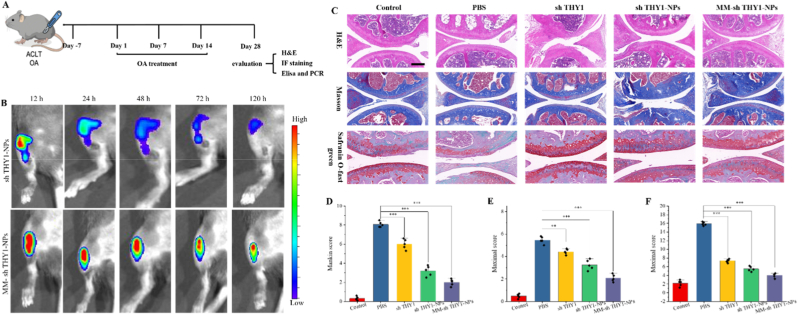
**In vivo targeting ability and therapeutic effects of MM-sh THY1-NPs on OA.** (A) Schematic illustration of the evaluation process for the therapeutic effect of MM-sh THY1-NPs on osteoarthritis. (B) Targeting of osteoarthritis and in vivo retention time after administration of sh THY1-NPs and MM-sh THY1-NPs in the knee. (C) H&E staining, Masson staining, and Safranin O-fast staining of rat knee joints. Scale bar, 50 μm. (D) Modified Mankin scores of rat knee joints. ∗∗P < 0.01; ∗∗∗P < 0.001, n = 3.

Previous experiments demonstrated the anti-inflammatory effects and cartilage microenvironment remodeling abilities of MM-sh THY1-NPs in vitro. To further evaluate the efficacy of MM-sh THY1-NPs in OA, ACLT-induced OA model was established. Cartilage degradation and inflammation, characteristic of OA pathology, are evident in the control group. As shown in Fig. 5A, after constructing the OA model in rats, PBS, sh THY1, sh THY1-NPs, and MM-sh THY1-NPs were injected into the knee joints, respectively, and treatment continued. On the 28th day post-operation, the rats were euthanized, and the joints were collected for further analysis.

To evaluate the therapeutic efficacy of MM-sh THY1-NPs in vivo, the targeting and retention time of NPs post-injection were assessed using dye assays. As depicted in Fig. 5B, sh THY1-NPs and MM-sh THY1-NPs accumulated in the knee joint within 12 h. After 24 h, the fluorescence intensity in the shTHY1-NPs group gradually decreased, while the fluorescence intensity in the MM-shTHY1-NPs group remained until 120 h. The prolonged intra-articular retention of MM-shTHY1-NPs is likely attributable to the combined effects of macrophage membrane-mediated biomimetic “self-like” recognition, which may reduce rapid phagocytic clearance, and the intrinsic joint affinity of the HA backbone, which may further enhance local adhesion/retention through CD44-related interactions within the inflamed joint microenvironment. This is attributed to the modification of macrophage membranes, allowing NPs to remain longer at the site of OA.

Further histological assessments using HE staining, Masson staining, and Safranin O-Fast Green staining were performed to evaluate changes in cartilage tissue. As shown in Fig. 5C, the control group had an intact and smooth cartilage surface with uniformly distributed cells and distinctly stained cartilage matrix. In comparison, the PBS group exhibited severe cartilage damage and degradation, a significantly thinner cartilage layer, and reduced cell numbers with cartilage matrix degradation, indicative of typical OA pathology. The sh THY1 and sh THY1-NPs groups also showed cartilage wear and cartilage matrix loss. In contrast, the MM-sh THY1-NPs group had an intact and smooth cartilage surface with uniform cell distribution and no cartilage matrix degradation, and the cartilage ECM staining was significantly better than other groups. Additionally, the modified Mankin score was used to assess cartilage damage in each group. As shown in Fig. 5D, the MM-sh THY1-NPs group had significantly lower Mankin scores than the other treatment groups, indicating that intra-articular injection of MM-sh THY1-NPs could improve OA cartilage damage.

### 3.6 Mechanism of MM-sh THY1-NPs in alleviating In vivo OA

**Fig. 6:**
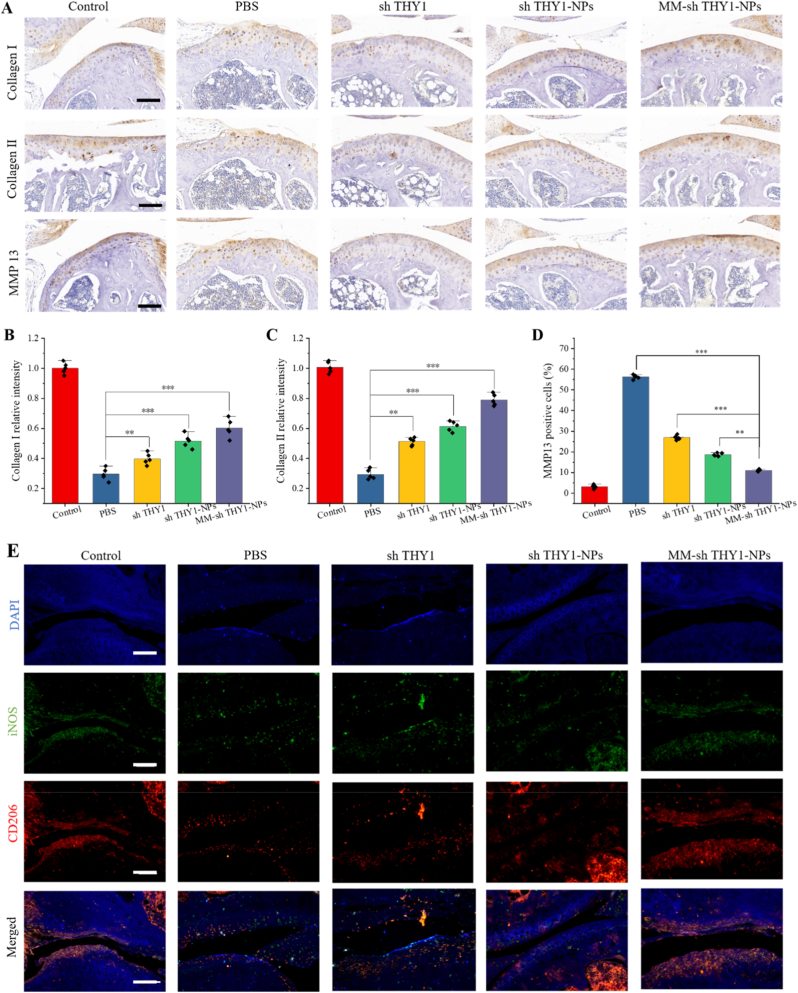
**Mechanism of MM-sh THY1-NPs in alleviating OA in vivo.** (A) IHC staining of collagen (Col) and MMP13 in OA rat cartilage. Scale bar, 100 μm. (B) Quantitative analysis of collagen I, (C) collagen II, and (D) MMP13 fluorescence intensity in knee joint cartilage regions. (E) Immunostaining of pro-inflammatory M1 biomarker (iNOS; green) and anti-inflammatory M2 biomarker (CD206; red) in rat knee joint sections after treatment with different materials. ∗∗P < 0.01; ∗∗∗P < 0.001, n = 3. Scale bar, 50 μm.

Immunohistochemistry was used to evaluate the damage and repair of cartilage in each group. As shown in Fig. 6A–D, compared to the control group, the expression of Col-I and Col-II significantly increased and MMP-13 expression significantly increased in the PBS, sh THY1, sh THY1-NPs, and MM-sh THY1-NPs groups. In contrast, the MM-sh THY1-NPs group had higher expression levels of Col-I and Col-II, which were significantly higher than other treatment groups (P < 0.05). MMP-13 expression in the MM-sh THY1-NPs group was also significantly lower than in the PBS, sh THY1, and sh THY1-NPs groups (P < 0.05). These in vivo findings further suggest that MM-shTHY1-NPs shift the cartilage microenvironment from a degradative catabolic state toward a reparative anabolic state, as reflected by reduced MMP13 expression and restored Collagen II production, which is consistent with the observed macrophage phenotype transition in the OA synovium.

To further analyze the mechanism by MM-sh THY1-NPs alleviate OA, immunohistochemistry (IHC) was employed to detect macrophage phenotypes. Immunofluorescence (IF) assays using iNOS and CD206 were performed to analyze macrophage types (Fig. 6E). Quantitative analysis (Fig. S11) showed that the fluorescence intensity of iNOS significantly increased in the PBS group, indicating that macrophages were stimulated to polarize into the M1 phenotype, while the fluorescence intensity of CD206 did not differ significantly from the control group. Subsequently, treatment with sh THY1, sh THY1-NPs, and MM-sh THY1-NPs significantly reduced the fluorescence intensity of iNOS and increased the fluorescence intensity of CD206. These results suggest that MM-sh THY1-NPs promote the polarization of M1 to M2 macrophages in the OA synovium. Although sh THY1 has a limited impact on enhancing the retention of nanoparticles, macrophage membrane-modified NPs can induce active uptake by macrophages, thereby providing optimal M1 to M2 polarization effects.

Additionally, the expression of related inflammatory factors was evaluated after treatment in each group. The expression of TNF-α was significantly upregulated in the PBS group (Fig. S12). However, this upregulation was reversed to varying degrees after treatment with different NPs, with the most significant inhibition observed in the MM-sh THY1-NPs treatment group. These experimental results support and verify the efficacy of MM-sh THY1-NPs in regulating macrophage polarization, highlighting their potential as a treatment for OA.

To evaluate the systemic safety of MM-shTHY1-NPs in vivo, major organs and blood samples were collected from rats at the end of the treatment cycle. H&E staining of the heart, liver, spleen, lung, and kidney revealed no discernible pathological abnormalities, including inflammatory infiltration, necrosis, or structural damage, in any group (Fig. S13). In addition, serum biochemical analysis showed that alanine aminotransferase (ALT) and aspartate aminotransferase (AST), as indicators of liver function, as well as blood urea nitrogen (BUN) and creatinine (CREA), as indicators of renal function, all remained within normal physiological ranges, with no significant intergroup differences. Collectively, these results demonstrate the favorable in vivo biocompatibility and low systemic toxicity of MM-shTHY1-NPs after intra-articular administration.

To further evaluate therapeutic efficacy from a functional perspective, we additionally assessed mechanical pain threshold and joint range of motion (ROM) in the different experimental groups. As shown in Fig. S14, OA induction markedly reduced the mechanical pain threshold and impaired joint mobility. In contrast, treatment with MM-shTHY1-NPs significantly restored pain threshold and improved ROM, showing the most pronounced functional recovery among the tested formulations. These findings further support the beneficial effect of MM-shTHY1-NPs on OA progression at the behavioral and functional levels.

## 4 Conclusion

OA is a highly prevalent degenerative disease in the elderly, characterized mainly by cartilage degradation and destruction. In recent years, RNAi-based nucleic acid delivery strategies have shown encouraging potential in OA treatment. The key to gene therapy is selecting an excellent gene carrier to deliver the target gene to the target cells, enabling it to perform its biological functions. Based on our previous studies, knocking down THY1 gene expression in vivo can alleviate OA progression. Therefore, we designed a macrophage-biomimetic gene delivery system where ROS-responsive polymer HA-SA-SD is coated with macrophage membranes, enabling active targeting of inflammatory tissues. Our materials provide a cell function-driven strategy that can target inflammatory sites without additional targeting molecules. Once targeted to the inflammatory site, MM-sh THY1-NPs, with their ROS-responsive properties, release sh THY1 at the inflammation site, thereby alleviating OA. In vitro and in vivo experiments demonstrated that MM-shTHY1-NPs suppress THY1-associated inflammatory activation in synovial fibroblasts, interrupt fibroblast–macrophage inflammatory crosstalk, promote macrophage repolarization toward the M2 phenotype, and ultimately alleviate OA progression. Thus, this biomimetic macrophage system holds great potential for further application.

## CRediT authorship contribution statement

**Xinyue Hu:** Conceptualization, Funding acquisition, Investigation, Methodology, Writing – original draft. **Zhuang Li:** Conceptualization, Investigation, Methodology, Writing – original draft. **Xiaofei Li:** Investigation, Methodology. **Lingxiao Zhang:** Conceptualization, Funding acquisition, Project administration, Supervision, Writing – review & editing. **Yaqing Zhang:** Conceptualization, Funding acquisition, Project administration, Supervision, Writing – review & editing.

## Declaration of competing interest

The authors declare the following financial interests/personal relationships which may be considered as potential competing interests: Given the role of Lingxiao Zhang as guest editor, had no involvement in the peer review of this article and had no access to information regarding its peer review. Full responsibility for the editorial process for this article was delegated to another journal editor. If there are other authors, they declare that they have no known competing financial interests or personal relationships that could have appeared to influence the work reported in this paper.

## Data Availability

Data will be made available on request.
