# Supplementary material for: A biomimetic nanoplatform enables shTHY1-mediated immunomodulation and cartilage remodeling for osteoarthritis therapy
Source: Mater Today Bio. 2026 Apr 21;38:103145. doi: 10.1016/j.mtbio.2026.103145 (PMC13134023; doi:10.1016/j.mtbio.2026.103145)
Supplement: Multimedia component 1 [file mmc1.docx]

***Supporting information for***

**A biomimetic nanoplatform enables shTHY1-mediated immunomodulation and cartilage remodeling for osteoarthritis therapy**

*Xinyue Hu^1, 2^, Zhuang Li^2^, Xiaofei Li ^2,3^, Linxiao Zhang^4*^, Yaqing Zhang^1*^*

^1^ Department of Pediatric Orthopedics, Xin Hua Hospital Affiliated to Shanghai Jiao Tong University School of Medicine, Shanghai, P. R. China.

^2^ School of Medicine, Southeast University, Nanjing, Jiangsu, P. R. China.

^3^ The Orthopedics Department, Jinhua Municipal Centeral Hospital,Jinhua Hospital of Zhejiang University Medical College, Jinhua, Zhejiang Province, P. R. China.

^4^ Interdisciplinary Nanoscience Center, Aarhus University, Aarhus C, 8000, Denmark.

**Corresponding author:**

***Yaqing Zhang, *E*-mail:** [zhangyaqingmed@163.com](mailto:zhangyaqingmed@163.com)

** Lingxiao Zhang, ***E*-mail:** [zhanglx@inano.au.dk](mailto:zhanglx@inano.au.dk)

**Figure S1-S6**

**Table S1**


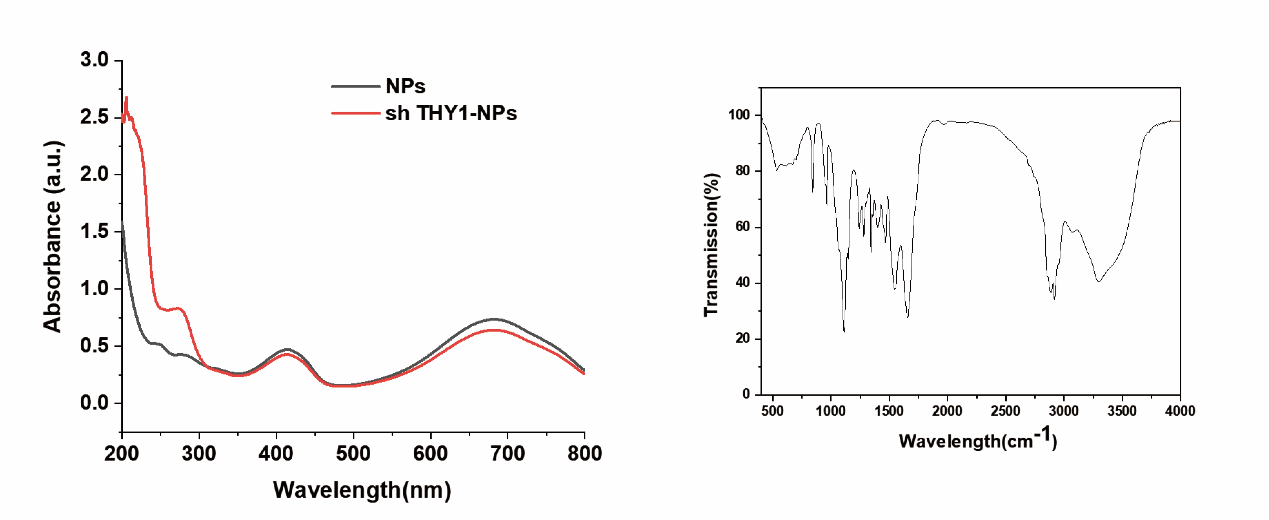


**Figure S1.** **UV-Vis and FTIR spectra of the NPs.**


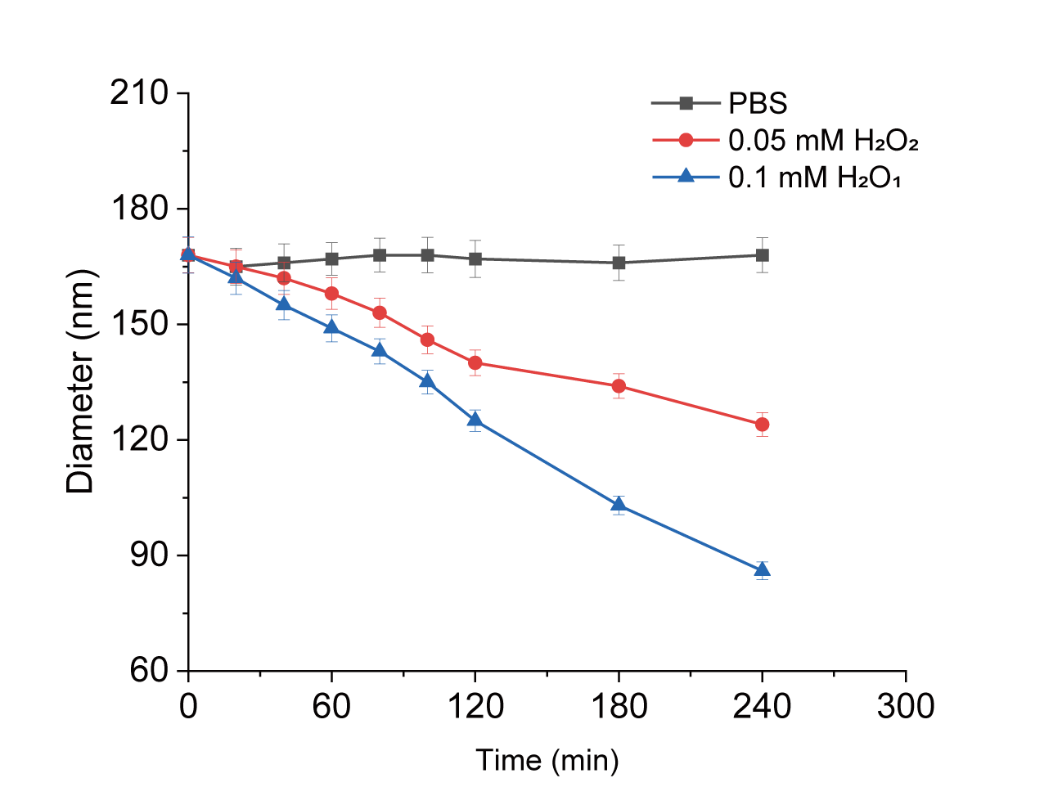


**Figure S2.** Time-dependent diameter size of shTHY1-NPs at different H_2_O_2_ concentrations (0.50 mM and 0.1 mM).


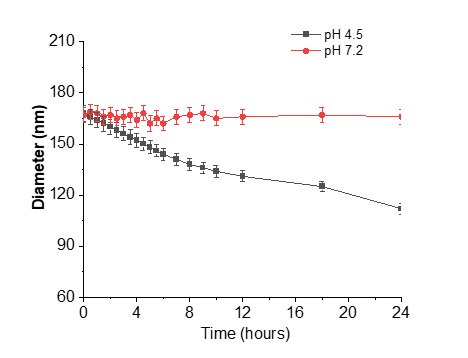


**Figure S3.** Time-dependent diameter size of shTHY1-NPs at different pH values.


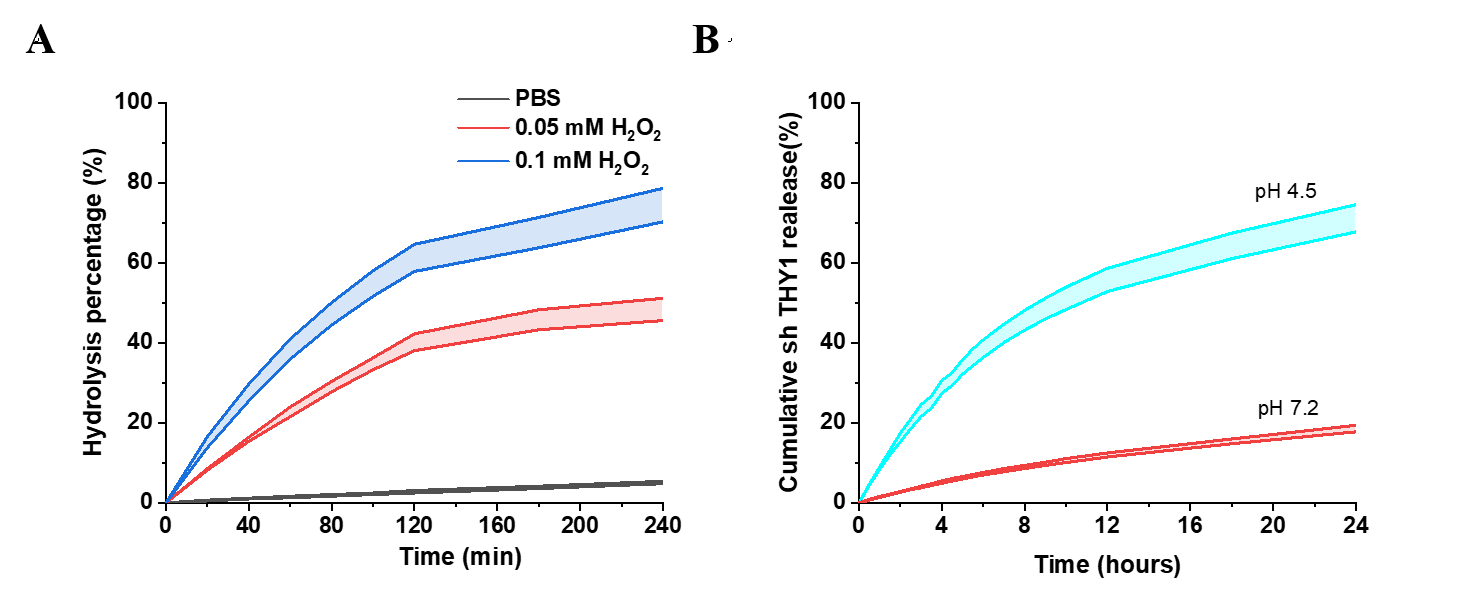


**Figure S4.** Microenvironment-responsive hydrolysis and pH-triggered shTHY1 release of MM-shTHY1-NPs. (A) Hydrolysis profiles of MM-shTHY1-NPs under different H2O2 concentrations (0, 0.1, and 0.5 mM) over time. (B) In vitro cumulative release profile of shTHY1 from MM-shTHY1-NPs at different pH values (pH 7.2 and pH 4.5).


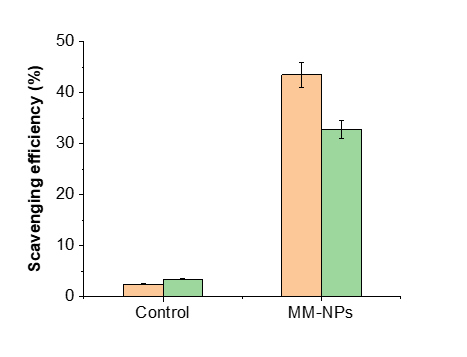


**Figure S5.** Quantitative evaluation of cytokine-scavenging capacity of macrophage membrane-coated nanoparticles.


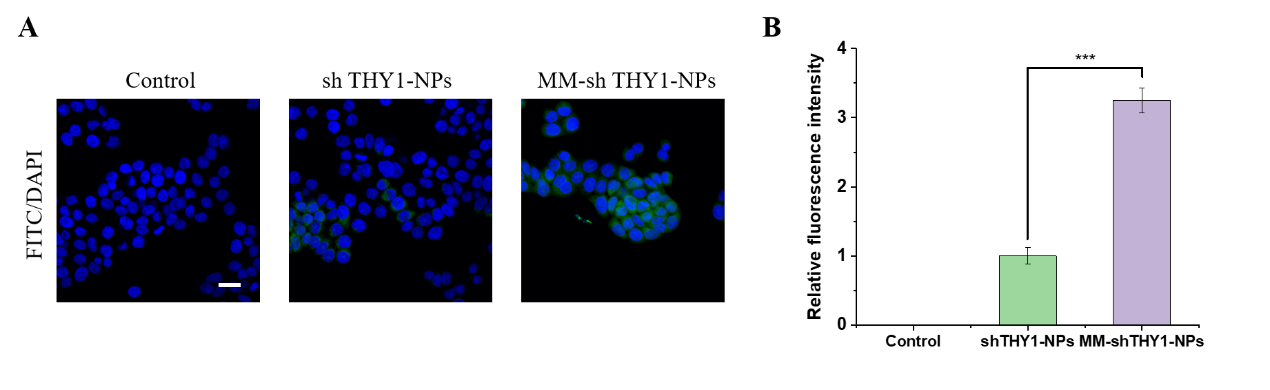


**Figure S6.** Comparative cellular uptake analysis of different nanoparticle formulations in macrophages. (A) Representative confocal images and (B) quantitative fluorescence analysis of macrophages incubated with different FITC-labeled formulations.


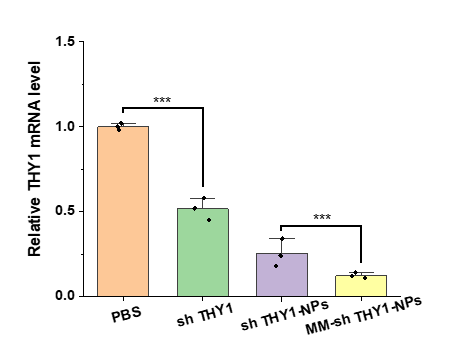


**Figure S7.** THY1 mRNA knockdown efficiency in macrophages after different treatments.


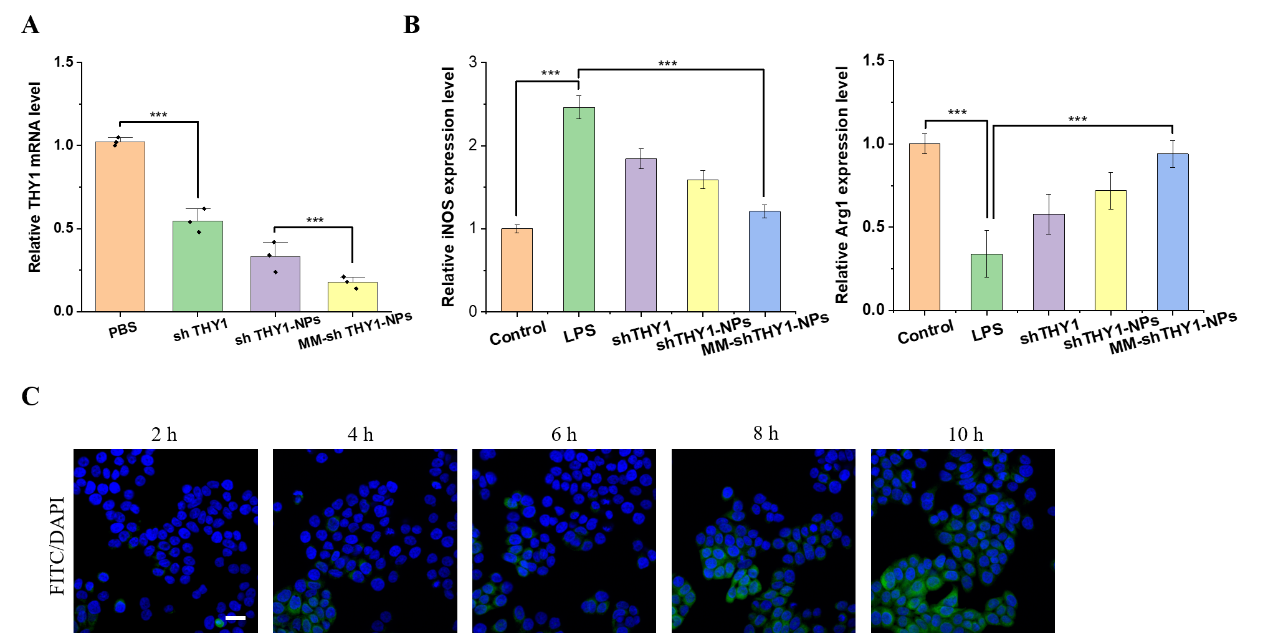


**Figure S8.** THY1 silencing in synovial fibroblasts and its downstream effects on macrophage polarization-related markers. (A) Relative THY1 mRNA expression in synovial fibroblasts after treatment with PBS, free shTHY1, shTHY1-NPs, or MM-shTHY1-NPs. (B) Relative expression levels of iNOS and Arg1 under inflammatory stimulation after the indicated treatments. ***P < 0.001. (C) LSCM images at different time points of synovial fibroblasts co-cultured with FITC-labeled NPs.


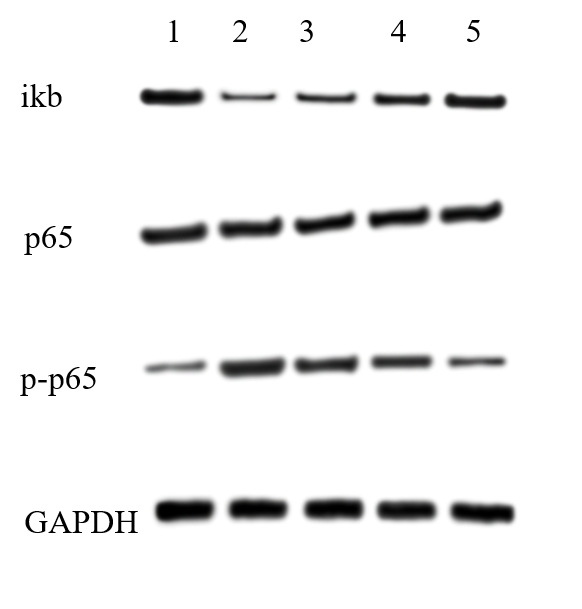


**Figure S9.** Western blot analysis of NF-κB-related signaling proteins in macrophages after different treatments. (1, Control; 2, LPS; 3, shTHY1; 4, shTHY1-NPs; 5, MM-shTHY1-NPs)


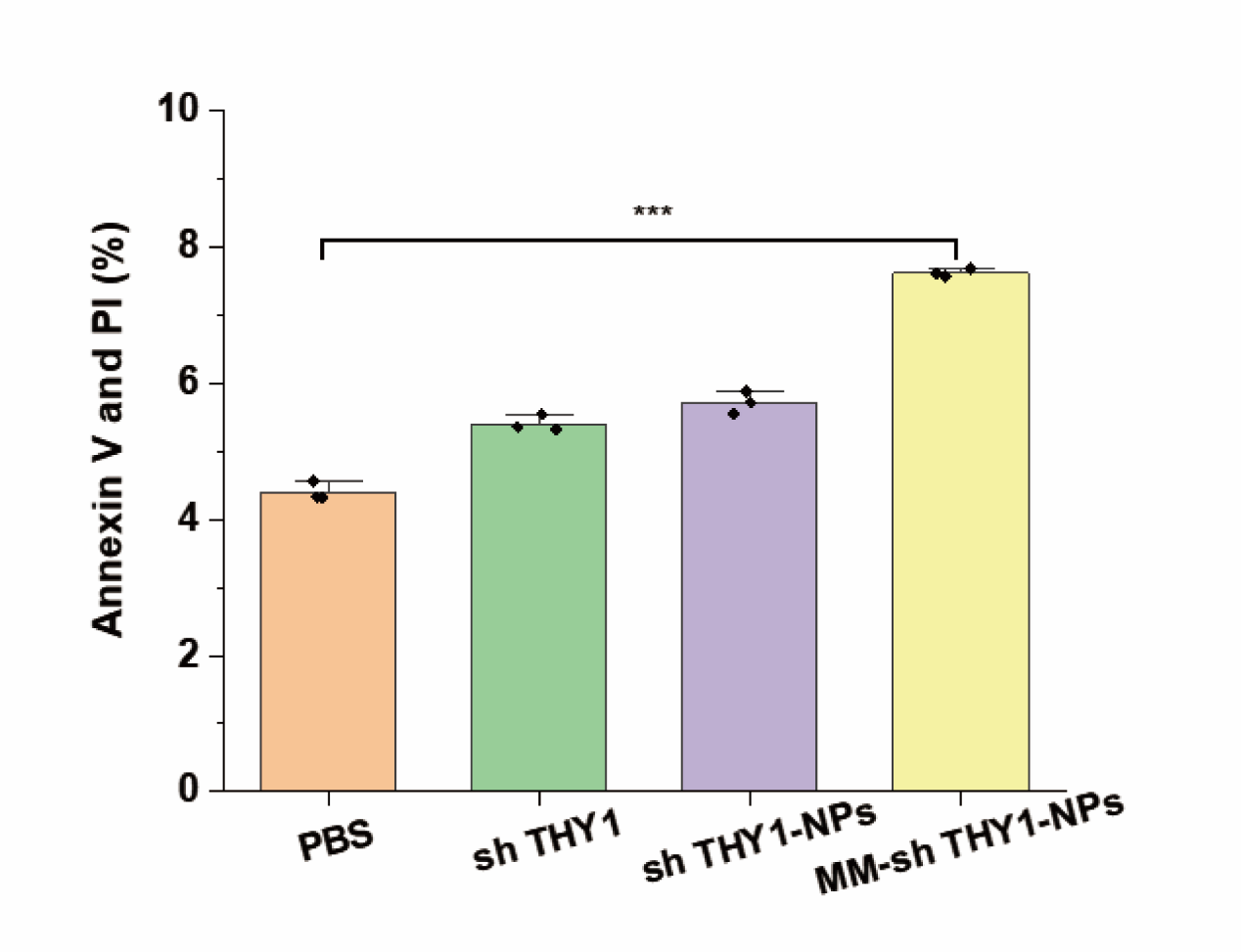


**Figure S10. Quantitative analysis of cell apoptosis.**


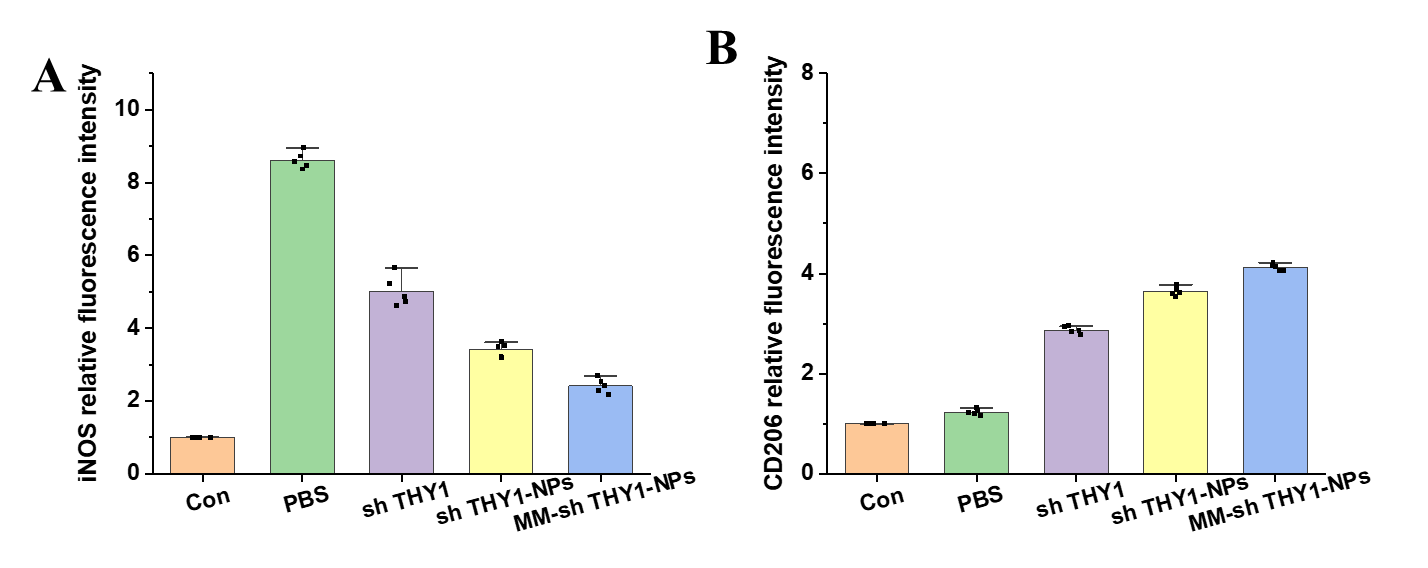


**Figure S11.** Quantitative analysis of (A) iNOS and (B) CD206 under various conditions. n = 3.


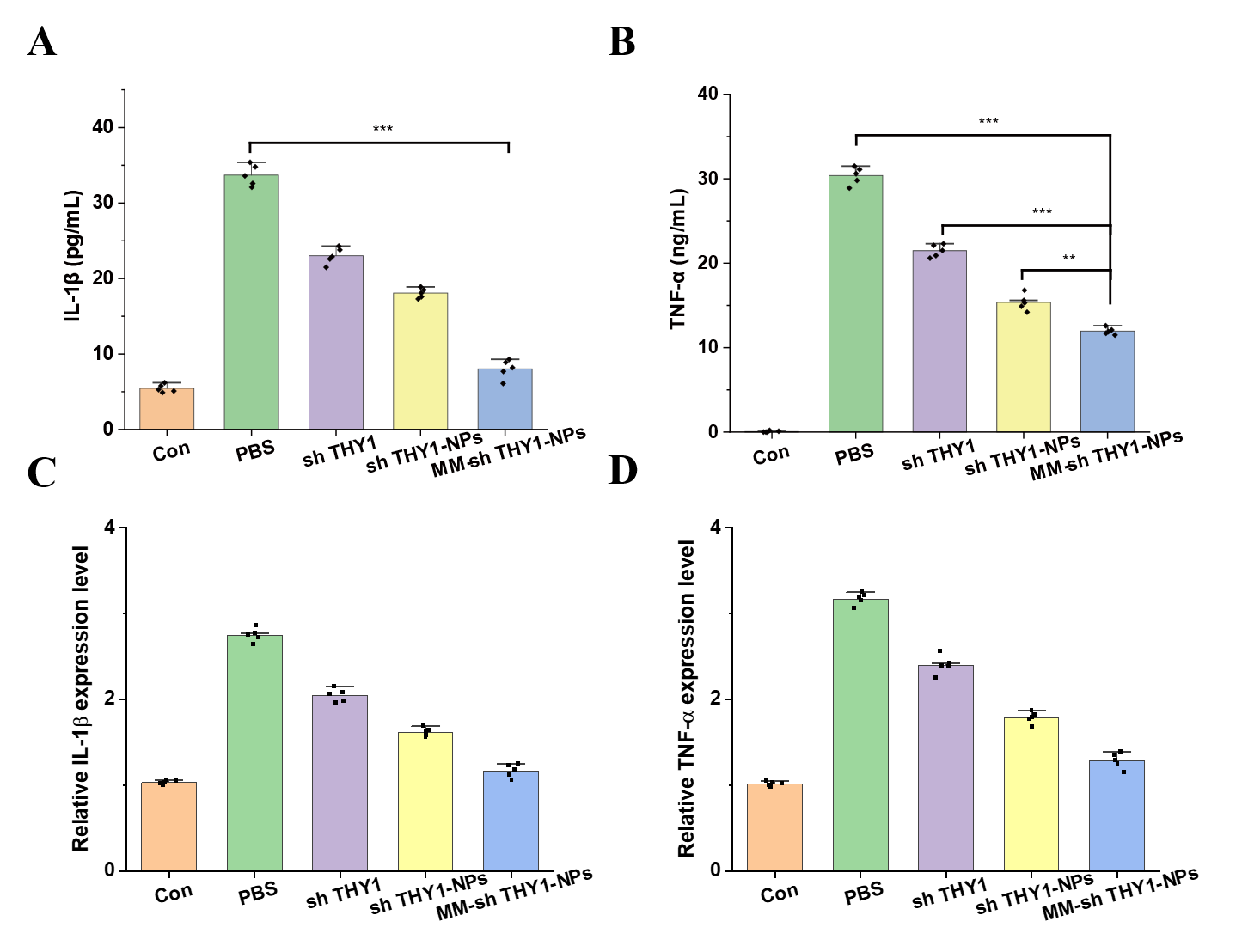


**Figure S12.** ELISA and qPCR quantification of IL-1β and TNF-α after different treatments. (A, B) ELISA measurements of IL-1β (A) and TNF-α (B) following the indicated treatments. (C, D) qPCR quantification of IL-1β (C) and TNF-α (D) mRNA levels after the same treatments. (n = 5). *P < 0.05; **P < 0.01; ***P < 0.001.


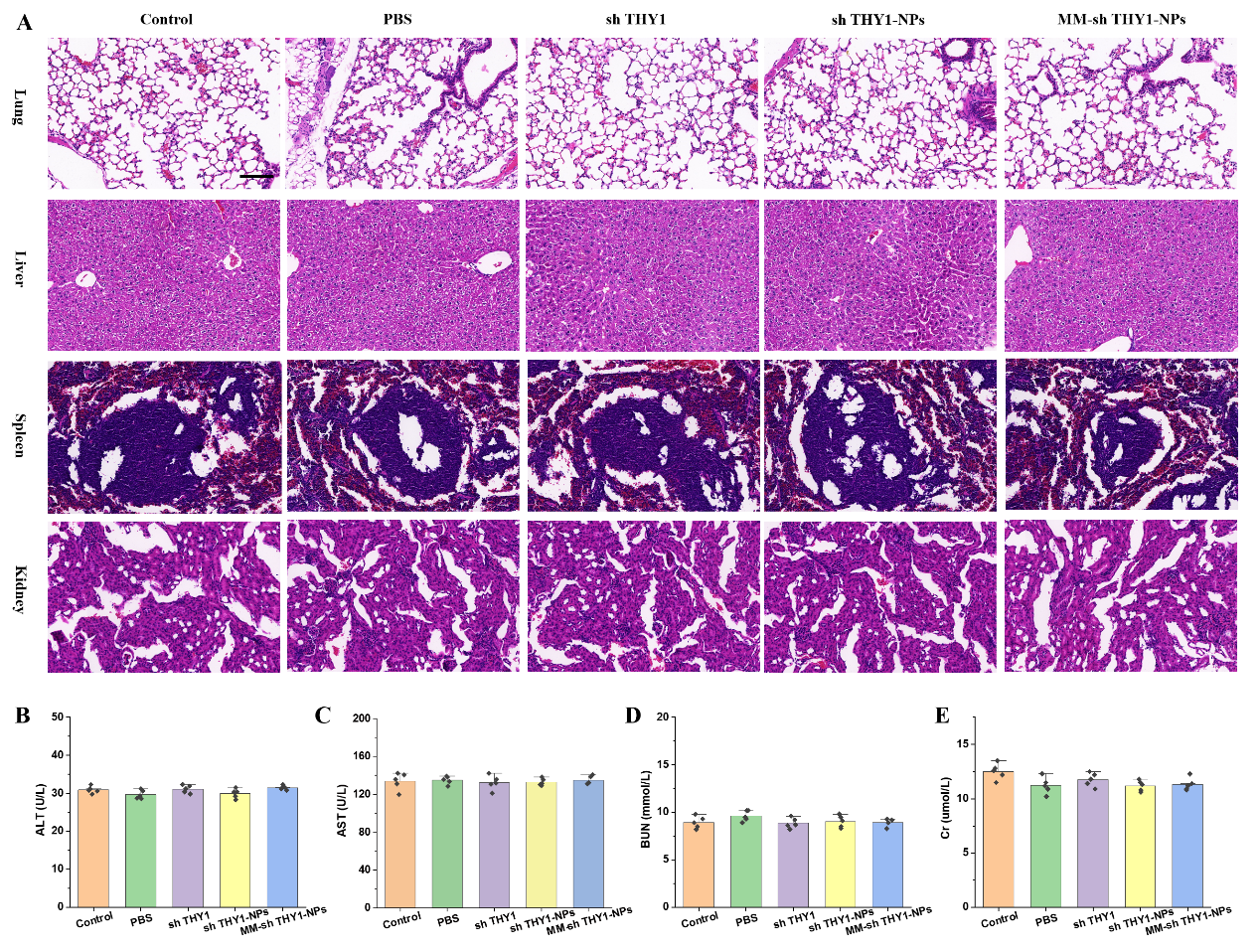


**Figure S13. Biocompatibility analysis.** (A) Hematoxylin and eosin (H&E) staining of major organs of mice in different treatment groups. (B-E) Hematological analysis of major organ function toxicity in mice. (B) Alanine aminotransferase (ALT): Liver function indicator; (C) Aspartate aminotransferase (AST): Liver function indicator; (D) Blood urea nitrogen (BUN): Kidney function indicator; (E) Creatine kinase (CK): Heart function indicator. Scale bar, 100 μm.


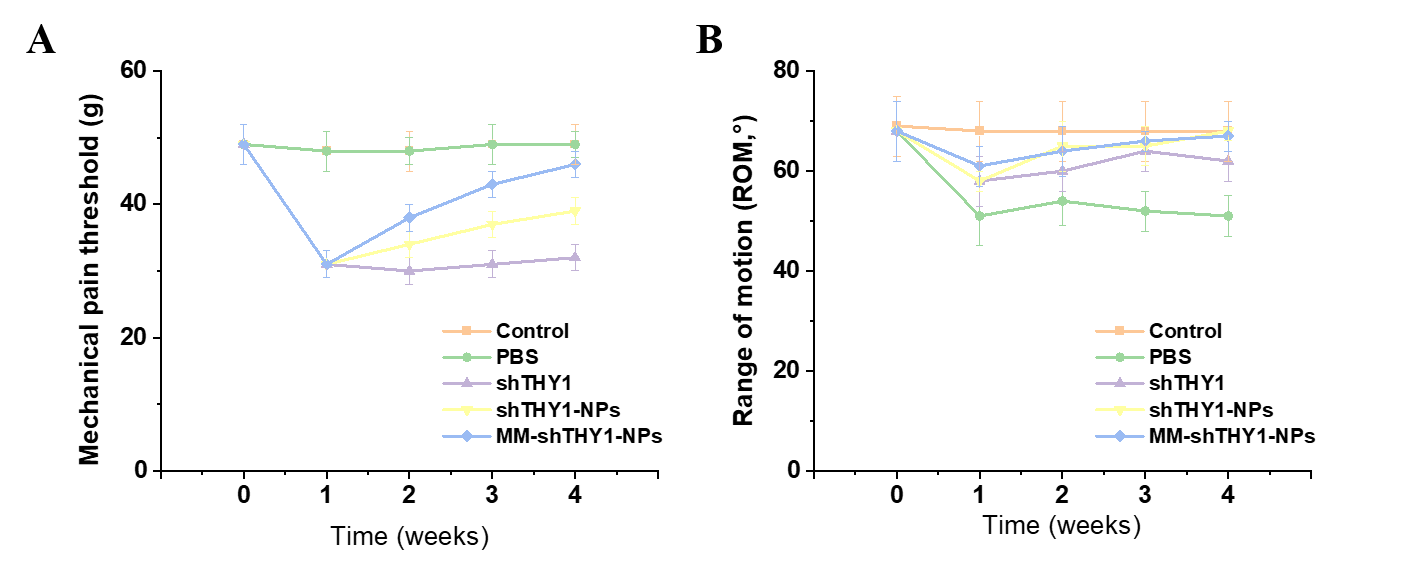


**Figure S14. Functional evaluation of therapeutic efficacy in the OA model.** (A) Mechanical pain threshold in different experimental groups. (B) Joint range of motion (ROM) in different experimental groups.

**Tab. S1.** qPCR primer sequences

| Primer | Forward primer | Reverse primer |
| --- | --- | --- |
| TNF-α | CCAGGAGAAAGTCAGCCTCCT | TCATACCAGGGCTTGAGCTCA |
| IL-1β | CACCTCTCAAGCAGAGCACAG | GGGGTTCCATGGTGAAGTCAAC |
| MMP13 | TGATGGACCTTCTGGTCTTCTGG | CATCCACATGGTTGGGAAGTTCT |
| GAPDH | GGACAATGGTGAAGGTCGGTGTGAAC | CAGCCGTGAGTGGAGTCATACTG |
